# An Effective Strategy for a Whole-Cell Biosensor Based on Putative Effector Interaction Site of the Regulatory DmpR Protein

**DOI:** 10.1371/journal.pone.0043527

**Published:** 2012-08-24

**Authors:** Saurabh Gupta, Mritunjay Saxena, Neeru Saini, Mahmooduzzafar, Rita Kumar, Anil Kumar

**Affiliations:** 1 Institute of Genomics and Integrative Biology, Mall Road, Delhi, India; 2 National Institute of Immunology, New Delhi, India; 3 Jamia Hamdard University, Hamdard Nagar, New Delhi, India; University of South Florida College of Medicine, United States of America

## Abstract

**Introduction and Rationale:**

The detection of bioavailable phenol is a very important issue in environmental and human hazard assessment. Despite modest developments recently, there is a stern need for development of novel biosensors with high sensitivity for priority phenol pollutants. DmpR (Dimethyl phenol regulatory protein), an NtrC-like regulatory protein for the phenol degradation of *Pseudomonas* sp. strain CF600, represents an attractive biosensor regimen. Thus, we sought to design a novel biosensor by modifying the phenol detection capacity of DmpR by using mutagenic PCR.

**Methods:**

Binding sites of ‘A’ domain of DmpR were predicted by LIGSITE, and molecular docking was performed by using GOLD to identify the regions where phenol may interact with DmpR. Total five point mutations, one single at position 42 (Phe-to-Leu), two double at 140 (Asp-to-Glu) and 143 (Gln-to-Leu), and two double at L113M (Leu-to- Met) and D116A (Asp-to- Ala) were created in DmpR by site-directed mutagenesis to construct the reporter plasmids pRLuc42R, pRLuc140p143R, and pRLuc113p116R, respectively. Luciferase assays were performed to measure the activity of *luc* gene in the presence of phenol and its derivatives, while RT-PCR was used to check the expression of *luc* gene in the presence of phenol.

**Results:**

Only pRLuc42R and pRLuc113p116R showed positive responses to phenolic effectors. The lowest detectable concentration of phenol was 0.5 µM (0.047 mg/L), 0.1 µM for 2, 4-dimethylphenol and 2-nitrophenol, 10 µM for 2, 4, 6-trichlorophenol and 2-chlorophenol, 100 µM for 2, 4-dichlorophenol, 0.01 µM for 4-nitrophenol, and 1 µM for o-cresol. These concentrations were measured by modified luciferase assay within 3 hrs compared to 6–7 hrs in previous studies. Importantly, increased expression of luciferase gene of pRLuc42R was observed by RT-PCR.

**Conclusions:**

The present study offers an effective strategy to design a quick and sensitive biosensor for phenol by constructing recombinant bacteria having DmpR gene.

## Introduction

Phenol and its organic compounds are the most important environmental pollutants at the global level that have been released into the environment in substantial quantities by natural events and industrial activities. Phenol is toxic, carcinogenic, mutagenic, and teratogenic even in low concentration and is mentioned in the list of priority pollutants of U.S. Environmental Protection Agency [1].

The detection of bioavailable phenol is a very important issue in environmental and human hazard assessment studies. Currently, chromatography coupled to the mass spectrometer detection is used for the analysis of phenol [2]. However, these methods are not used extensively because of being cost intensive and the chemicals used in these processes may harm the surrounding environment [3]. Biosensors can provide rapid measurement without labor-intensive and time-consuming sample preparation and apparently allow for a promising way to assess the biologically available phenol in the environment. Previously, different biosensors for phenol have been described such as the amperometric [4], [5], enzymatic [6], and optical biosensor [7].

Microbes offer attractive targets for the construction of biosensors for monitoring the status of the environment. Whole-cell microbial sensors have become one of the latest approaches of molecular tools in environmental monitoring [8]. Microorganisms, for their low cost, lifespan, and range of suitable pH and temperatures, have been widely used as the biosensing recognition elements in the construction of biosensors [9] Currently available whole cell biosensors have many advantages as they can provide an inexpensive and simple way of determining contaminants. As they are living organisms, they give a more accurate response on the toxicity of different compounds. Some stress-induced biosensors report the mutagenic effects of samples with great sensitivity. Biosensors are unexcelled in gene expression and physiological study of bacteria in complex environments. One of the greatest limitations of whole-cell biosensor development is the availability of strong promoters that respond only to relevant stimuli. To surmount this obstacle, more knowledge on gene regulatory networks in bacteria is needed [10]. Numerous whole cell biosensors for phenol have been developed in which different reporter and regulator genes with various lowest detection ranges of up to 291.4 mg/L (highest) [11] and 0.082 mg/L (lowest) [12] were reported. However, there is always a need for less cumbersome and sensitive approaches to monitor phenol levels. A different approach to construct a microbial biosensor is to connect a strictly regulated promoter sequence to a sensitive reporter gene was reported previously [13]. The ability of the bacteria to survive in a contaminated environment is usually based on a genetically encoded resistance system, the expression of which is regulated very precisely.

DmpR (Di methyl phenol regulatory protein), the product of the *Pseudomonas sp.* strain CF600 DmpR gene [14], [15], mediates the expression of Dmp operon to allow growth on simple phenols. Transcription from Pdmp, the promoter of the Dmp operon, is activated when DmpR detects the presence of an inducing phenol [15]. Previous studies of the sensory ‘A’ domain may provide some insights regarding interaction between phenol and Dmp protein [16]. The natural interaction of DmpR with a subset of phenols suggested that the modification of its sensor domain might result in protein which can detect a broader range of phenolic derivatives [17]. Till date, there is no crystal structure for the DmpR protein. However, a threading model for N-terminal ‘A’ domain of DmpR has been reported [18], [19].

The aim of this study was to develop a new whole-cell luminescence-based bacterial sensor for highly selective and sensitive detection of bioavailable phenol in the environment. We accomplished this by choosing the DmpR gene with Po/Pr operator/promoter gene from *Pseudomonas sp.* CF600 as a receptor for phenol as an analyte and *luc* genes from pGL3 vector (Promega, USA) as a reporter. Pr promoter of DmpR *w*as identified as a σ_70_ dependent promoter that is regulated by σ_54_ dependent Po promoter [20]. Previous mutational studies [15], [21], [22] were based on the random analysis in DmpR gene. In this study, mutant residues were predicted by docking tools instead of being chosen randomly. Mutant forms of DmpR with altered effector specificity were made by a dock-based site directed mutagenesis method. Three mutant plasmids pRLuc42R, pRLuc140p143R and pRLuc113p116R were constructed by creating mutations in the DmpR gene. The pRLuc42R contains one single mutation F42L (Phenylalanine at 42→Leucine), whereas the pRLuc140p143R and pRLuc113p116R possess two point mutations each (a) D140E (Aspartic acid at 140→Glutamic acid), and (b) Q143L (Glutamine at 143→Leucine); (a) L113M (Leucine at 113→Methionine) and (b) D116A (Aspartic acid at 116→Alanine). Mutant with the single mutation F42L (Phe at 42→Leu) was particularly interesting since it showed an increased response to the phenol inducer. ‘A’ domain of the DmpR is responsible for sensing the phenol and its derivative. Mutations were induced in this region by computational prediction and validated by mutagenic PCR in the wet lab.

## Experimental Procedures

### 2.1. Bacterial Strains and Culture Conditions

*Escherichia coli* strain DH5α (hsdR, recA, thi-1, relA1, gyrA96) [23] was used for the maintenance of plasmids. *Pseudomonas* sp. CF600 [24] was obtained from Dr. Victoria Shingler (Umea, Sweden) and grown in Nutrient broth at 37°C. The *E. coli* DH5α harbouring the plasmids was cultured at 37°C in Luria Bertani media (1% tryptone, 0.5% yeast extract and 0.5% NaCl) containing 100 mg/ml ampicillin. The list of used strains and vectors is given in Table 1.

**Table 1:** Bacterial strains and plasmids.

Strains or Plasmids	Relevant Genotype	Reference
DH5α	supE44,DlacU169(/80lacZ DM15), hsdR17, endA1, recA, thi-1,relA1, gyrA96	Hanahan (1983)
Pseudomonas sp.CF600	Utilizes and degrade Phenol	Shingler et al (1989)
pGL3-basic	Ap^r^, promoter less cloning vector	Promega
pGL3-promoter	Ap^r^, SV40 promoter cloning vector	Promega
pRLuc (DmpR/Pr/Po)	pGL3-basic vector containingwild type DmpR/Pr and Po promoter	This study
pRLuc42R	pGL3-basic vector containing DmpR/Pr and Po promoter andmutation at 42 amino acid	This study
pR140p143R	pGL3-basic vector containing DmpR/Pr and Po promoter andmutation at 140 and 143 amino acid	This study
pRLuc113p116R	pGL3-basic vector containing DmpR/Pr and Po promoter andmutation at 113 and 116 amino acid	This study

### 2.2. Construction of pRLuc42R Biosensor

**Figure 1:**
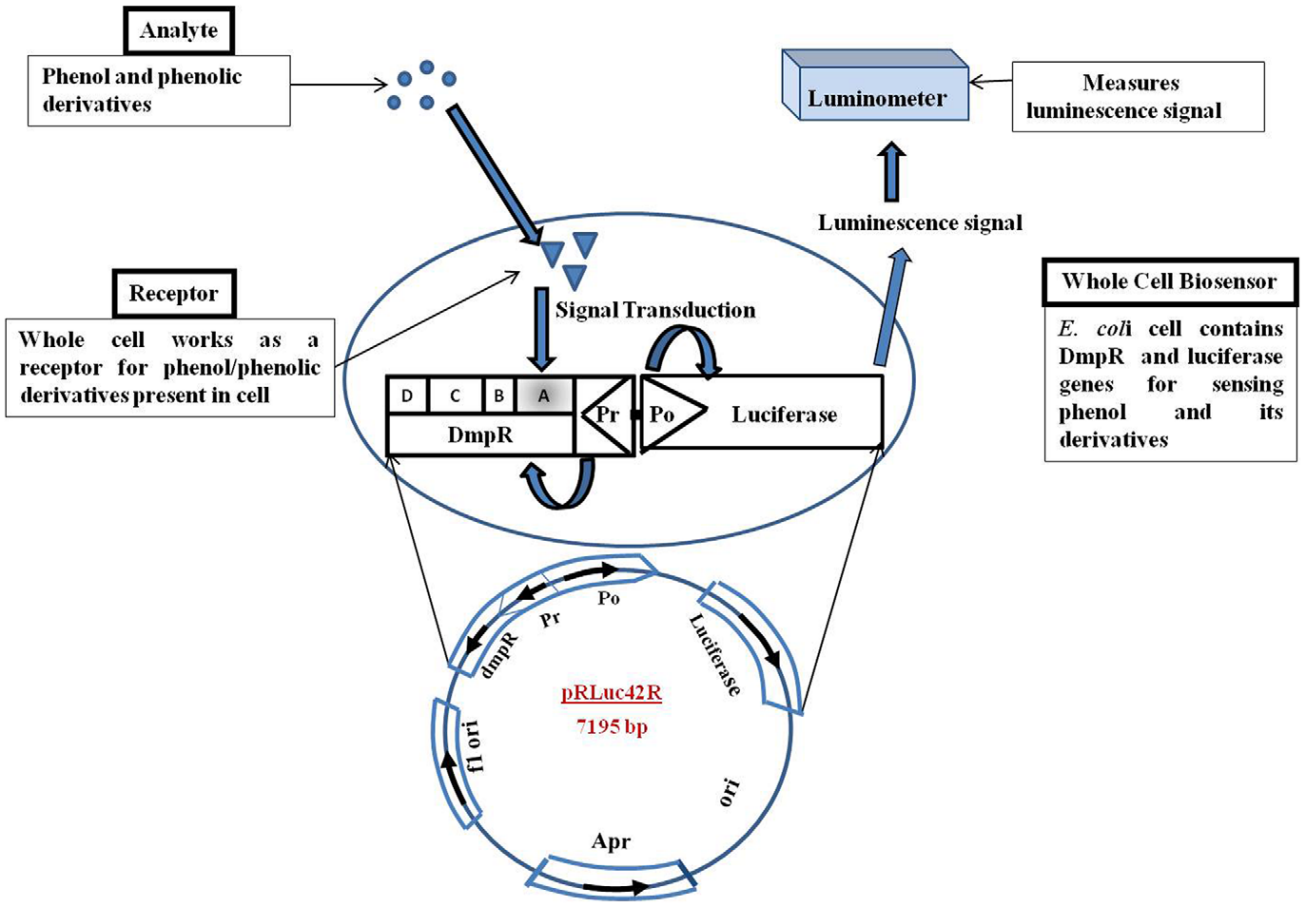
Schematic illustration of sensing principle of whole cell biosensor (pRLuc42R) to detect phenol at glance.

Whole cell biosensor (pRLuc42R) sensing principle is shown in Figure 1.

#### 2.2.1. Plasmid isolation, cloning and PCR

Genomic DNA of *Pseudomonas* sp. CF600 was prepared by using a Genomic DNA isolation kit (RBC, Taiwan). Plasmids were isolated with a Qiagen spin column kit (CA, USA). The expression vectors, pGL3 basic and pGL3 promoter vector were purchased from Promega (WI, USA). Cloning, ligation, and transformation were carried out by using standard techniques [25]. PCR was performed by using forward primer 5-CGATCGATGCTCAGCTCGAGGCCAGGTTAGGCGTAGGACGCA-3 and a reverse primer, 5-GCTAGCATCGGTATAAGCTTCGCACACGGATGTAACGAGTGA-3, with XhoI and HindIII sites at the ends. PCR conditions were as follows: 95°C for 5 min followed by 33 cycles of 95°C for 45 s, 55°C for 45 s, and 72°C for 1 min, followed by 72°C for 7 min. *Taq* DNA polymerase and deoxynucleoside triphosphates were from New England Biolabs (USA).

#### 2.2.2. Site-directed mutagenesis and cloning

**Figure 2:**
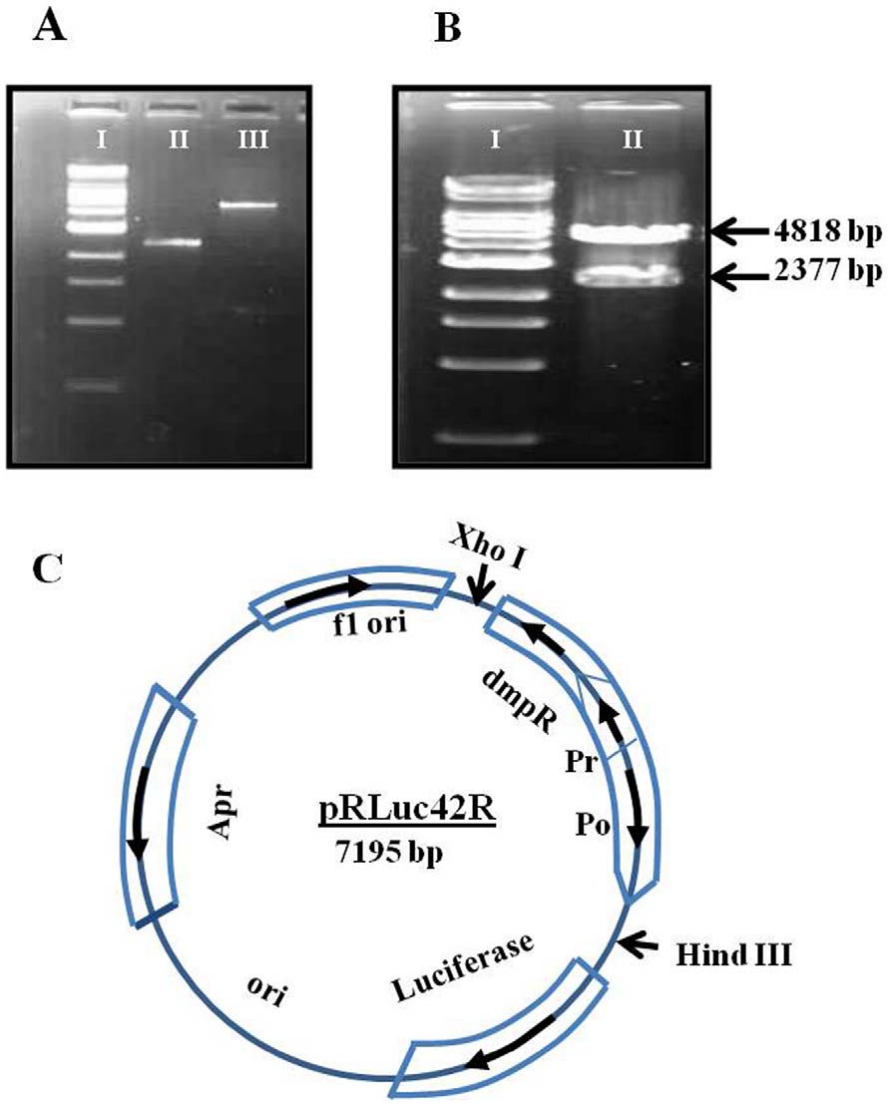
Construction of plasmid pRLuc42R. The DmpR (1.692 kb) and its promoter (Pr) and operator (Po) were cloned from *Pseudomonas sp.* CF600. DNA segment was digested with XhoI and Hind III and introduced upstream of the luciferase gene in the pGL3 basic expression vector. The arrows indicate the transcription or processing direction for genes. (a) Digested and gel purified pGL3 vector and Gel Purified DmpR+Po Promoter for Ligation (b) Double digestion of pRLuc42R. (c) Vector drawing of pRLuc42R Fig (a) I-500 bp ladder, II-2377 bp (insert), III-4818 bp (vector) Fig (b) I-500 bp ladder, II-Double digested pRLuc42R.

Mutagenesis was performed by using the mutagenic PCR [26]. The primers used for site-directed mutagenesis are as follows, (a) for F42L (to generate pRLuc42R) forward primer: TGTTGCTGCAGCTTTCAGCGATGG and reverse primer: GCCATCGCTGAAAGCTGCAGCAA (b) for D140E and Q143L (to generate pRLuc140p143R) forward Primer: ACCGAACTGGGGCTGATGCA and reverse primer: TGCATCAGCCCCAGTTCGGT (c) for L113M and D116A (to generate pRLuc113p116R) forward Primer: ACCGAGATGGATATCGCCAAGGAA and reverse primer: TTCCTTGGCGATATCCATCTCGGT (Altered codons are underlined). Mutagenic PCR product containing mutated DmpR (1692 bp) and the promoters, Pr and Po fragments (total 2377 bp) were gel-isolated and digested with XhoI and HindIII (New England Biolabs, USA), and introduced upstream of the firefly Luciferase (*luc*) in the pGL3 basic expression vector to generate approximately 7 kb of pRLuc42R plasmid (Figure 2). The same method was used to generate pRLuc, pRLuc140p143R and pRLuc113p116R. Plasmids were transformed into *E. coli* DH5α by the CaCl_2_ transformation method. The nucleotide sequences of the pRLuc42R were determined by DNA sequencing from the TCGA (The Centre for Genomic Application, New Delhi, India).

### 2.3. Proposed Binding Sites

LIGSITE algorithm [27] was used to predict the active site of ‘A’ DmpR model for phenol and phenolic derivatives. Thereafter the binding pocket which were predicted by LIGSITE, were given an input to GOLD software for docking analysis.

### 2.4. Treatment of Phenolic Compounds and Activity Assays for Luciferase

Various phenolic compounds (Sigma and Aldrich Chemical Co., MO, USA) were dissolved in ethanol and added to media at final concentrations ranging from 10 nM to 1 mM. The *E. coli* DH5α cells harboring the plasmid were grown overnight at 37°C in 4 ml of LB medium containing 100 mg/ml ampicillin. Cells were sub-cultured and grown to the log phase of 0.2 in OD at 600 nm [28] followed by supplementation with phenolic compounds at various concentrations. After 1 h, 50 µl of the cells were withdrawn, and kept frozen at −70°C with the addition of 5 µl of 1 M KH_2_PO_4_ and 20 mM EDTA (pH 7.8). Cells were lysed by incubating at room temperature for 10 min with the addition of 150 µl of lysis solution (1.25 mg/ml lysozyme, 2.5 mg/ml BSA, 1X CCLR) (Promega, WI, USA). Supernatants were obtained by centrifugation. For the luciferase activity, 20 µl of supernatant was mixed with 20 µl of firefly luciferin solution (Promega, WI, USA). The bioluminescence was measured for 20 s by luminometer Berthold Detection System (Germany).

### 2.5. Calculations

**Figure 3:**
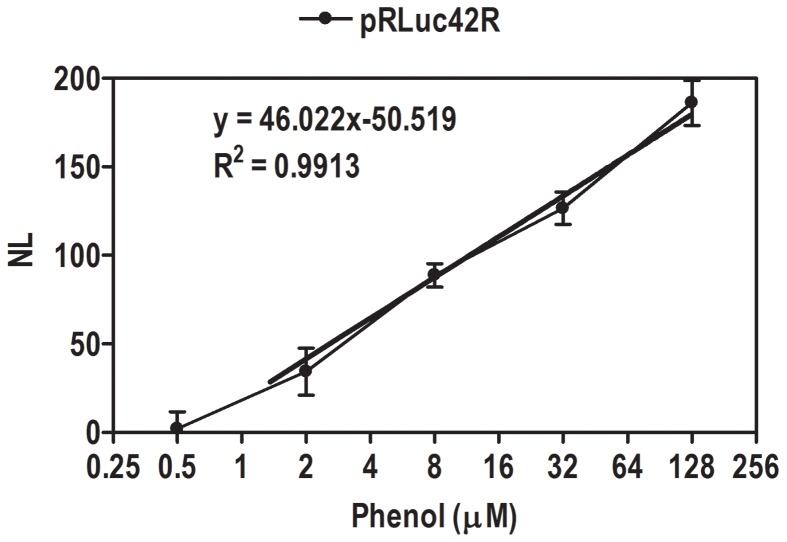
Lineariry curve is presented as phenol concentration v/s NL value of luminescence. Firefly luciferase activities expressed in *E. coli* cells with pRLuc42R grown at different concentration of phenol. The pRLuc42R strain showed highly sensitive response at a low concentration of 0.5 µM. R^2^ = 0.9913. NL - Normalised luminescence, µM - Micro molar concentration.

Induction of the pRLuc42R sensor by phenol and phenolic derivatives was expressed as normalised luminescence (NL) calculated as follows [9]:-.
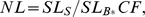
Where *SL_S_* is the luminescence of the sensor in the dilution of the phenolic compound, *SL_B_* is the background luminescence of the sensor bacteria, and *CF* is the correction factor.



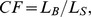
Where *L_B_* is the background luminescence of the control bacteria, and *L_S_* is the luminescence of the control bacteria in phenol/phenolic compound. The detection limit for the sensor bacteria for different phenols was defined as a concentration of the compound which induced the sensor twice above the background level, i.e. NL ≥2. Linearity curve for phenol induction is presented in Figure 3.

### 2.6. Chemical Analysis of Phenolic Compounds

Chemical analysis of phenolic compounds was performed according to the standard method recommended by American Public Health Association [29]. Synthetic water was filtered through a 0.2 µm membrane (Sartorius, Germany) and then serially diluted in 1 ml volume. Subsequently, 25 ml of 0.5 N NH_4_OH, 10 ml of 2% 4-aminoantipyrine (Sigma, MO, USA), and 10 ml of 8% K_3_Fe(CN)_6_ (Sigma, MO, USA) were added in order and mixed thoroughly. OD at 500 nm was measured.

### 2.7. RNA Isolation and Real Time PCR

Total RNA was isolated by RNeasy Mini Kit (Qiagen, USA) from pRLuc42R, pRLuc, pRLuc140p143R, pRLuc113p116R and pGL3-promoter vector (Promega, USA) containing *E. coli* cells treated with or without phenol. Total RNA was converted into cDNA by cDNA synthesis kit (Roche, USA). The RT-PCR (Model 7500, Applied Biosystems, CA, USA) was performed in a MicroAmp Optical 96 well reaction plate (Applied Biosystems, CA, USA). The primers for *luc* and DmpR gene were designed by using Primer Express 1.0 (Applied Biosystems, CA, USA). The forward and reverse primers for luc gene were 5′-TGGAGAGCAACTGCATAAGG-3′ and 5′-CGTTTCATAGCTTCTGCCAA-3′, respectively, while the forward and reverse primers for the DmpR gene were 5′-AAATCCAGCACTCCGATTTC-3′ and 5′-CCCAGGGTATTGACCATTTC-3′. The specificity of primers was examined under the standard PCR conditions prior to quantitation by real-time RT-PCR. The primers have been synthesized by TCGA (New Delhi, India). Each RT-PCR reaction contained the following: 1× SYBR Green Master Mix (containing assay buffer, dNTPs, and Polymerase enzyme), primer pair (1 µM), and cDNA, in 25 µl of reaction volume. Real time RT-PCR was carried out for 30 min at 60°C following initial 2 min incubation at 50°C to prevent carryover reactions. The reaction was terminated by dissociation step by heating at 95°C for 15 s, 60°C for 1 min and finally 95°C for 15 s. The PCR amplification was then performed for 40 cycles with each cycle at 95°C for 15 s and 60°C for 1 min. All reactions were carried out in triplicate using the ABI 7500 Real Time PCR. The threshold cycle, C_T_, values were averaged from the values obtained from each reaction, and the mRNA expression was calculated as a relative level of expression.

### 2.8 Statistical Analysis

The data represents mean±SD of three independent experiments. The data was analyzed by one-way ANOVA followed by Newman-Keuls Multiple Comparison Test. The p value of <0.05 represents statistical significance in different groups.

## Results and Discussion

Among the most abundant environmental pollutants, the aromatic compounds (such as phenol or phenolic compounds) are of major concern because of their persistence and toxicity. The environmental problems caused by such coherent pollutants have increased the demand for the development of sensitive pollutant and toxicity detection methods. The fusion of reporter genes to promoters that are induced when cells are exposed to chemicals is one promising approach that has been used to formulate biosensor for such application [30].

### 3.1. Construction of Whole Cell Biosensor pRLuc42R

For confirmation of DmpR protein binding to phenol, *E. coli* whole cell luminescence biosensor (pRLuc42R) was constructed. The mutated sequence (mutation was suggested by docking and computational study) of 2377 bp (includes DmpR, Pr and Po) were inserted upstream of *luc* in pGL3 basic expression vector to generate pRLuc42R as a final construct to form whole cell biosensor for phenol. Reporter enzymes such as chloramphenicol acetyltransferase, firefly luciferase, and β-galactosidase from *E. coli* are frequently used for studies of gene regulation, gene activity, and expression in eukaryotic cells [31]. Firefly luciferase, in particular, has been extensively applied in molecular and cell biology studies because of its negligible background, less incubation time, high sensitivity, and the relative simplicity of the assay [32], [33]. A previous study [34] has shown that firefly luciferase can also be used for analysis in prokaryotic cells. Therefore, firefly luciferase was used as a reporter in this study.

### 3.2. Active Site Prediction

In our previous study [35], we performed the docking procedure for phenol and N-DmpR model with GOLD by using both scoring functions (GOLD Score and Chemscore). The GOLD Fitness score and Chemscore for the selected phenolic compounds are shown (Table 2). In selected phenolic derivatives, phenol showed highest GOLD Fitness score of 33.42 whereas the lowest Chemscore was 19.98.

**Table 2:** The GOLD Fitness score, Chemscore for the selected phenolic derivatives (Ligand) are shown.

Ligand	Goldscore	Chemscore
Phenol	33.42	19.98
2,4-dimethylphenol	31.4	21.89
2-chlorophenolphenol	29.54	22.85
4-nitrolphenol	32.46	21.29

**Figure 4:**
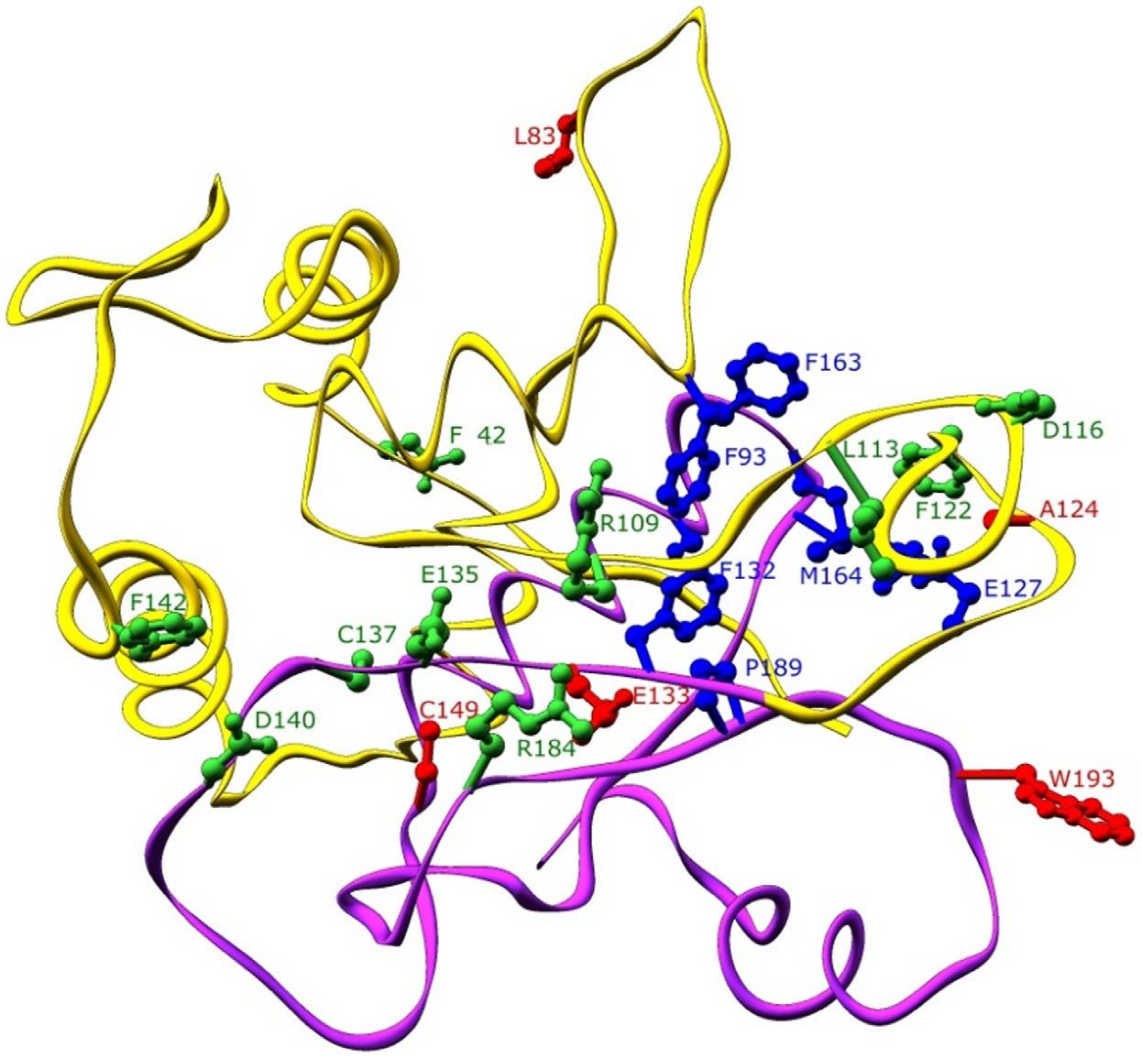
Experimentally mutated residues of N-terminal ‘A’ domain of DmpR. The N-DmpR model is shown in ribbon representation in yellow colour. V4R domain is shown in red colour. Side chains are represented in a ball and stick model. Mutated residues that negatively affect the aromatic effector response are shown in red colour. Mutated residues that positively increase detection of phenol derivatives are shown in green colour. Predicted active site residues are shown in blue. Phe 93, Phe 132, and Phe 163 residues also negatively affect the aromatic effector response present in the active sites. Image was produced by using PyMOL software (http://www.pymol.org).

Figure 4 shows the docked phenol with ‘A’ domain-DmpR model. Image was produced by using PyMOL software [36]. Experimental mutated residues and active site pockets (in blue colour) of N-DmpR are also shown in this figure. Predicted positive key catalytic residues are (in green colour) Phe 42, Asp 140, Arg109, Arg 184, Phe 122, Cys 137, Leu 113, Asp 116 etc; while negative catalytic residues are (in red colour) Cys 149, Glu 133, Ala124, Trp193, and Leu83.Out of these residues our study was based on mutations in Phe42, Asp140,Glu143, Leu 113, and Asp 116 respectively. Apparently, this figure shows that the predicted ligand binding site is located in and near the V4R domain of N-DmpR. Previously, It has been reported that the V4R domain is a small molecule binding domain, which may bind to hydrocarbons [37].

The current study shows the validation of mutation sites and altered amino acids, provided by random mutations in previous studies [38], [39]. Based on Gold/Chemscore, amino acid/acids were opted for site directed mutagenesis. Since there is no 3D structure available for DmpR till date, the only option for us was to predict mutations in the dry lab and then try to prove these in wet lab experiments.

### 3.3. Responses of pRLuc42R to Various Phenolic Compounds

**Figure 5:**
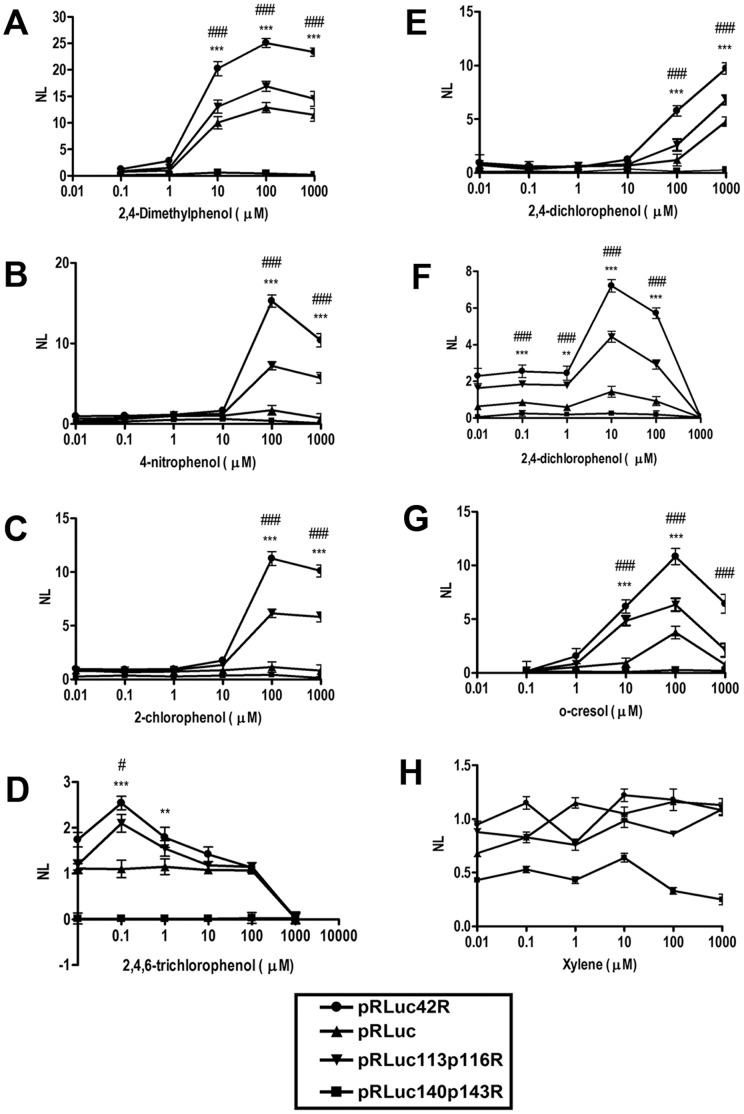
pRLuc42R exhibits superior luciferase activity compared with pRLuc and other mutants. Cells were grown without (m) or with the addition of various phenolic compounds ranging from 10 nM to 1 mM for 1 h in the case of cells with pRLuc42R. The activities were shown as the ratio of induced to non-induced activities. The four sets of data (pRLuc42R, pRLuc, pRLuc140p143R and pRLuc113p116R) are the average of three independent experiments. (A) 2,4-dimethylphenol (B) 2,4,6-trichlorophenol (C) 2-chlorophenol (D) 2-nitrophenol (E) 2, 4-dichlorophenol (F) 4-nitrophenol, (G) o-cresol, (H) Xylene. **p<0.01, ***p<0.001 compared with normalized luciferase activity of pRLuc; #p<0.05, ###p<0.001 compared with normalized luciferase activity of pRLuc113p116R; one-way ANOVA followed by Newman-Keuls Multiple Comparison Test.

The cells with pRLuc42R (Figure 5) showed very promising results with various phenolic compounds (p<0.001 compared with pRLuc, pRLuc113p116R, or pRLuc140p143R). Priority pollutant phenols were chosen for the testing including phenol, 2-chlorophenol, 2,4-dichlorophenol, 2,4,6-trichlorophenol, Pentachlorophenol, 4-chloro-3-ethylphenol, 2,4-dimethylphenol, 2-nitrophenol, 4-nitrophenol, 2,4-dinitrophenol, 2-methyl-4, 6-dinitrophenol, o-cresol, 4-chloro-3-ethylphenol, and xylene. Xylene was used as a negative control.

The effective compounds were phenol, 2-chlorophenol, 2,4-dichlorophenol, 2,4,6-trichlorophenol, 2,4-dimethylphenol, 2-nitrophenol, 4-nitrophenol, and cresol; and the minimum concentrations (in µM) for detection of these effective compounds were 10, 100, 10, 0.1, 0.1, 0.01, and 1, respectively. These are the minimum detection limits so far for these compounds [12], [15], [17], [31] (Table 3). The sensor is more sensitive to 4-nitrophenol (0.01 µM), 2-nitrophenol (0.1 µM) and 2,4-dimethylphenol (0.1 µM) than phenol (0.5 µM). This is an advantage since these phenolic derivatives, especially the 2,4-dimethylphenol was considered as the compound responsible for carcinogenic influence while nitro phenols can exert mutagenic influence [40].

**Table 3:** Comparative table for the existing bacterial biosensors for phenol.

S.No.	Description	pRLuc42R Phenol Biosensor (This study)	Shingler et al.(1994)	Wise and kuske (2000)	Park et al. (2003)	Leedjarv et al. (2006)
1.	Regulatory gene for Phenol+reporter gene	Mutated DmpR+luc	DmpR wt & DmpR-XylR hybrid +lux	DmpR+LacZ	CapR+Luc	DmpR+lux
2.	Wild Type/Mutant	Both	Both	Both	Wild type	Wild type
3.	Mutants (Positions)	Docking based at 42^nd^ position of A domain	Random	Random	NA	NA
	**Pollutants**					
	**Priority pollutant Phenols(USEPA)**	**Detection Limit**	**Detection Limit**	**Detection Limit**	**Detection Limit**	**Detection Limit**
I	Phenol	500 nM	3.2 µM	2.5 µM	10 µM	0.082 mg/l (871 nM)
II	2-chlorophenol	1 µM		2.5 µM by Mutant	100 µM	
III	2, 4-dichlorophenol	10 µM		25 µM by Mutant		
IV	2, 4, 6-trichlorophenol	0.1 µM		Not Detected		
V	Pentachlorophenol	Not detected		Not Detected		
VI	4-chloro-3-ethylphenol	100 µM		75 µM by Mutant		
VII	2, 4-dimethylphenol	0.1 µM		75 µM by Mutant		0.454 mg/l (3.71 µM)
VIII	2-nitrophenol	100 µM		75 µM by Mutant	100 µM	
IX	4-nitrophenol	10 µM		75 µM by Mutant		
X	2, 4-dinitrophenol	Not detected				
XI	2-methyl-4,6-dinitrophenol	Not detected		Not Detected		
	**Other Phenolic derivatives**					
A	Cresol & derivatives	10 µM	3.2 µM-2 mM		100 µM	
	**Non Phenolic derivatives**					
	Xylene	Not detected				

On the other hand, 2, 4-dinitrophenol, 2-methyl-4, 6-dinitrophenol, pentachlorophenol, 4-chloro-3-ethylphenol, and Xylene were not detected by the system. The detection range and maximum responses of luciferase activity varied according to the compounds tested. The strong bioluminescence responses were caused by phenol, 2,4-dimethyphenol, and 2-chlorophenol. It has been known that phenol and methyphenol compounds had strong effects on the regulatory proteins involved in phenol degradation. The effective concentration of phenolic compounds for the regulatory proteins is also one of the major concerns. However, only a single concentration of effectors, usually around 2–3 mM was used in the measurement for DmpR in previous reports [12, 15, 18, and 34]. In the present studies, phenolic compounds in a range from 0.01 mM to 1 mM were examined. These results suggested that the residue L42 (Leucine at 42), M113 (Methionine at 113) and A116 (Alanine at 116) might be involved in inducer binding while the inducer binding might not be occurring in case of E140 (Glutamic acid at 140) and L143 (Leucine at 143). It is noteworthy that our system shows very low concentration ranges of phenolic compounds for detection. This aspect of the bacterial biosensors described here could be useful in detecting phenolic compounds at lower concentrations.

### 3.4. Luciferase Assay and Real Time PCR

**Figure 6:**
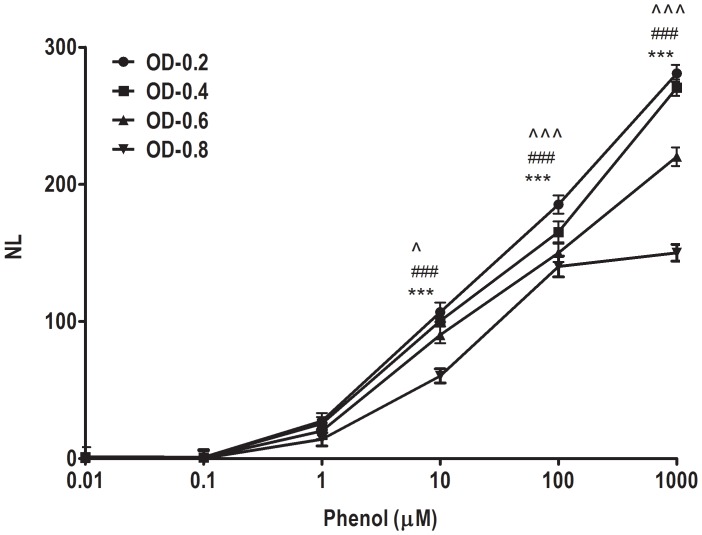
pRLuc42R firefly luciferase activities expressed in *E. coli* cells show enhanced sensitivity at low absorbance of 0.2. The data are the average of three independent experiments with standard deviations. NL - Normalised luminescence. ^?^p<0.05, ^???^p<0.001 compared with phenol + OD 0.4; ###p<0.001 compared with phenol + OD0.6; and ***p<0.001 compared with phenol + OD0.8. The data was analyzed by one-way ANOVA followed by Newman-Keuls Multiple Comparison Test.

*E. coli* cells harbouring pRLuc42R plasmid were grown with the addition of various concentrations of phenol, and their luciferase activities were measured. Luciferase shows stronger signals in almost all ranges of phenol and phenolic derivative concentration. The pattern of responses began to appear around 10 nM and continued to increase up to 1 mM. These results indicate that the DmpR/phenol complex binds to Po operator to activate luciferase genes most effectively, owing to the fact that the cloned DmpR is an activator gene implicated in the metabolism of phenol [14]. In addition, it was shown that the insect luciferase is stably expressed when they are grown to the log phase of 0.2 (instead of 0.3 or 0.4) in OD at 600 (p<0.001) (Figure 6). As a result, the time consumption for the assay was decreased by 2–3 hours, as compared to 6–7 h in previous studies [12], [34] which made it a relatively faster luciferase assay for phenol detection. The total time taken to complete the assay includes the incubation time for reaching the desired OD and induction with phenol. The quantitation of the luminescence is possible with a liquid scintillation counter, a luminometer, or even X-ray film.

**Figure 7:**
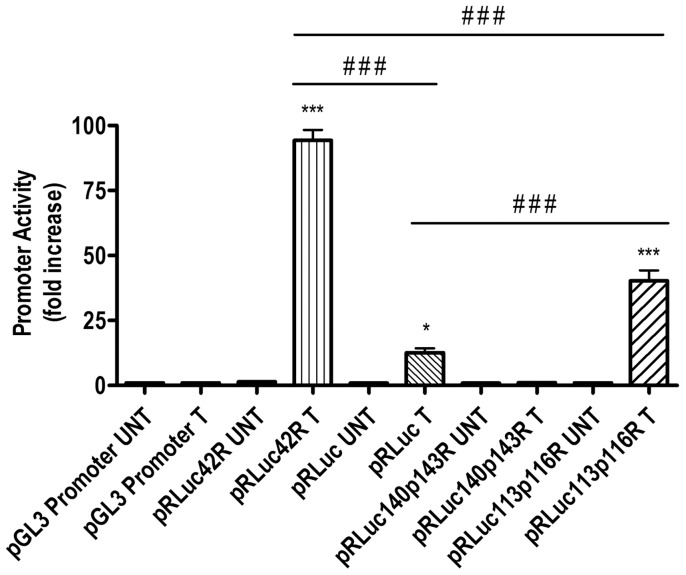
Phenol (1 **mM)-treated pGL3 promoter pRLuc42Rdemonstrates enhanced promoter activity.** Luciferase promoter activity of phenol-treated pRLuc42R, pRLuc, pRLuc140p143R, and pRLuc113p116R were determined by RT-PCR and presented as fold-increase. The data are the average of three independent experiments with standard deviations. *p<0.05, ***p<0.001 compared to respective (phenol)-untreated group; ###p<0.001 in comparisons as shown in figure. The data was analyzed by one-way ANOVA followed by Newman-Keuls Multiple Comparison Test.

Real time PCR was performed to confirm the responses to phenol with pRLuc42R, pRLuc and pRLuc140p143R. The threshold cycle (C_T_) values for expression of *luc* gene in pRLuc42R, pRLuc, pRLuc140p143R, pRLuc113p116R and pGL3 were 8.02±0.21, 10.80±0.63, 14.63±0.78, 9.24±0.27 and 14.64±0.43, respectively. The ΔC_T_ was calculated with reference to the pGL3 promoter vector. As a result, real time PCR shows approximately 7 folds and 100 folds increased expression of luciferase gene of pRLuc42R (by **2^−ΔΔ CT^** Method), when compared to unmodified system pRLuc and pRLuc140p143R, respectively. Folds change has been compared with 1 mM phenol treated and untreated cells of all construct (Figure 7). In luciferase assay, decrease in the response occurring at high concentration of phenolic compounds appears to be due to the toxic effect of phenolic compounds on the cells. It has been suggested that these phenolic compounds may interact with specific components of the electron transport chain, thus inhibiting electron transport and oxidative phosphorylation [41], [42]. Even though it cannot be excluded that cell viability may affect the total activities at high concentration of phenolic compounds, major increase in responses seem to occur in harmless or sub-lethal ranges. Taken together, the results indicate that the pRLuc42R protein has different properties in effecter specificity from other relevant regulators for catabolic pathways of phenolic compounds.

### 3.5. Assessment of Phenolic Compounds in Synthetic Water

It is shown above that *E. coli* cells with DmpR could be used as whole cell biosensor to monitor various pure phenolic compounds. The results show that this system can also detect phenolic compounds in synthetic water. The total amount of phenolic compounds in water measured by a chemical analysis exceeded the phenol amount obtained. The whole cell luminescent biosensor is similar to the chemical analysis method in a sense that they can detect the total phenolic compounds (data not shown). However, its results must be considered in a more complex way due to the differential effects of different phenolic derivatives. Moreover, the concentration of each phenolic compound in synthetic water might be achieved by further refined instrumental analysis.

The biosensor described here is very simple, economical and fast in measurement, possibly with sustaining sensitivity and reproducibility. It might reflect various aspects of pollutants such as bioavailability and toxicity during the measurement [43], [44]. So far the whole cell biosensors have offered unique alternative methods in identifying and quantifying phenol and its derivatives [12], [15], [18], [34]. However, the present sensor does not to detect some phenolic derivatives out of eleven priority pollutant phenols (http://www.epa.gov/) but this is capable in detection of seven priority pollutants phenol and o-cresol. In summary, our data shows that the DmpR and its promoters may be of use for the development of whole cell biosensor, to specifically monitor the contamination of phenolic compounds in the environment.

### Conclusions

Microbial biosensor is a simple, rapid, and effective analytical device that combines a biological sensing element with a transducer. As a sensing element, microorganism has not only advantages of easy manipulation and adaptation to the environment but also disadvantages of poor selectivity, low sensitivity and cytotoxicity. Here, we present one promising strategy to address the problems by tailoring microorganism through mutagenesis to the DmpR regulatory gene. Single mutant (pRLuc42R) was constructed at residues 42 of ‘A’ domain in the DmpR regulatory gene, using docking/computational studies. Replacement of amino acids resulted in positive effects on inducer responses. The pRLuc42R shows increased sensitivity. Thus, the mutagenesis method could open endless possibilities of microorganisms for excellent biosensing elements by overcoming the drawback of low sensitivity and cytotoxicity, eventually resulting in the development and application of microbial biosensors for prospective future advances.

## References

[1] Agency USEP website. Available at: http://www.epa.gov. Accessed 2012 July 27.

[2] Herchi W, Sakouhi F, Khaled S, Xiong Y, Boukhchina S, et al (2011) Characterisation of the glycerophospholipid fraction in flaxseed oil using liquid chromatography–mass spectrometry. Food Chemistry 129: 437–442. 10.1016/j.foodchem.2011.04.096 30634249

[3] Jiang HL, Tay JH, Tay ST (2002) Aggregation of immobilized activated sludge cells into aerobically grown microbial granules for the aerobic biodegradation of phenol. Lett Appl Microbiol 35: 439–445. 12390497 10.1046/j.1472-765x.2002.01217.x

[4] Silva LM, Salgado AM, Coelho MA (2011) Development of an amperometric biosensor for phenol detection. Environ Technol 32: 493–497. 21877529 10.1080/09593330.2010.504234

[5] Ceto X, Cespedes F, Pividori MI, Gutierrez JM, Del Valle M (2011) Resolution of phenolic antioxidant mixtures employing a voltammetric bio-electronic tongue. Analyst 137: 349–356. 22102984 10.1039/c1an15456g

[6] Yildiz HB, Castillo J, Guschin DA, Toppare L, Schuhmann W (2007) Phenol biosensor based on electrochemically controlled integration of tyrosinase in a redox polymer. Microchimica Acta 159: 27–34.

[7] Abdullah J, Ahmad M, Heng LY, Karuppiah N, Sidek H (2006) Chitosan-based tyrosinase optical phenol biosensor employing hybrid nafion/sol-gel silicate for MBTH immobilization. Talanta 70: 527–532. 18970803 10.1016/j.talanta.2005.12.061

[8] Yagi K (2007) Applications of whole-cell bacterial sensors in biotechnology and environmental science. Appl Microbiol Biotechnol 73: 1251–1258. 17111136 10.1007/s00253-006-0718-6

[9] Mulchandani B, Rogers KR (1998) Enzyme and Micriobial Biosensors: Techniques and Protocols. Humana Press: Totowa, NJ, USA.

[10] Liu X, Germaine KJ, Ryan D, Dowling DN (2010) Whole-cell fluorescent biosensors for bioavailability and biodegradation of polychlorinated biphenyls. Sensors (Basel) 10: 1377–1398. 22205873 10.3390/s100201377 PMC3244019

[11] Liu C, Yong D, Yu D, Dong S (2011) Cell-based biosensor for measurement of phenol and nitrophenols toxicity. Talanta 84: 766–770. 21482280 10.1016/j.talanta.2011.02.006

[12] Leedjarv A, Ivask A, Virta M, Kahru A (2006) Analysis of bioavailable phenols from natural samples by recombinant luminescent bacterial sensors. Chemosphere 64: 1910–1919. 16581105 10.1016/j.chemosphere.2006.01.026

[13] Peng Z, Yan Y, Xu Y, Takeo M, Yu H, et al (2010) Improvement of an E. coli bioreporter for monitoring trace amounts of phenol by deletion of the inducible sigma54-dependent promoter. Biotechnol Lett 32: 1265–1270. 20533077 10.1007/s10529-010-0317-6

[14] Shingler V, Bartilson M, Moore T (1993) Cloning and nucleotide sequence of the gene encoding the positive regulator (DmpR) of the phenol catabolic pathway encoded by pVI150 and identification of DmpR as a member of the NtrC family of transcriptional activators. J Bacteriol 175: 1596–1604. 8449869 10.1128/jb.175.6.1596-1604.1993 PMC203952

[15] Shingler V, Moore T (1994) Sensing of aromatic compounds by the DmpR transcriptional activator of phenol-catabolising Pseudomonas sp. strain CF600. J Bacteriol 176: 1555–1560. 8132448 10.1128/jb.176.6.1555-1560.1994 PMC205239

[16] O’Neill E, Ng LC, Sze CC, Shingler V (1998) Aromatic ligand binding and intramolecular signalling of the phenol-responsive sigma54-dependent regulator DmpR. Mol Microbiol 28: 131–141. 9593302 10.1046/j.1365-2958.1998.00780.x

[17] Schaffer AA, Aravind L, Madden TL, Shavirin S, Spouge JL, et al (2001) Improving the accuracy of PSI-BLAST protein database searches with composition-based statistics and other refinements. Nucleic Acids Res 29: 2994–3005. 11452024 10.1093/nar/29.14.2994 PMC55814

[18] Devos D, Garmendia J, de Lorenzo V, Valencia A (2002) Deciphering the action of aromatic effectors on the prokaryotic enhancer-binding protein XylR: a structural model of its N-terminal domain. Environ Microbiol 4: 29–41. 11966823 10.1046/j.1462-2920.2002.00265.x

[19] Cabrera C, Svergun D, Shingler V, Sauer UH (n.d.) Solution properties of DmpR – A phenol sensing, sigma-54 dependent transcription co-activator and member of the AAA + superfamily of proteins. Journal of Structural Biology: 371–372. Available at: http://hasyweb.desy.de/science/annual_reports/2006_report/part2/contrib/73/18327.pdf. Accessed 2012 July 27.

[20] Johansson LUM, Solera D, Bernardo LMD, Moscoso JA, Shingler V (2008) σ54-RNA polymerase controls σ70-dependent transcription from a non-overlapping divergent promoter. Mol Microbiol 70: 709–723. 18786144 10.1111/j.1365-2958.2008.06440.x

[21] Wise AA, Kuske CR (2007) Detection of phenols using engineered bacteria. USA Patent no. 7,303,894.

[22] Wise AA, Kuske CR (2000) Generation of novel bacterial regulatory proteins that detect priority pollutant phenols. Appl Environ Microbiol 66: 163–169. 10618218 10.1128/aem.66.1.163-169.2000 PMC91800

[23] Hanahan D (1983) Studies on transformation of Escherichia coli with plasmids. J Mol Biol 166: 557–580. 6345791 10.1016/s0022-2836(83)80284-8

[24] Shingler V, Franklin FC, Tsuda M, Holroyd D, Bagdasarian M (1989) Molecular analysis of a plasmid-encoded phenol hydroxylase from Pseudomonas CF600. J Gen Microbiol 135: 1083–1092. 2559941 10.1099/00221287-135-5-1083

[25] Sambrook J, Russell DW (2001) Molecular Cloning: A Laboratory Manual New York.: Cold spring Harbor.

[26] Vogne C, Bisht H, Arias S, Fraile S, Lal R, et al (2011) Characterisation of the putative effector interaction site of the regulatory HbpR protein from Pseudomonas azelaica by site-directed mutagenesis. PLoS One 6: e16539. 21379585 10.1371/journal.pone.0016539 PMC3040749

[27] Hendlich M, Rippmann F, Barnickel G (1997) LIGSITE: automatic and efficient detection of potential small molecule-binding sites in proteins. J Mol Graph Model 15: 359–363, 389. 10.1016/s1093-3263(98)00002-3 9704298

[28] Willardson BM, Wilkins JF, Rand TA, Schupp JM, Hill KK (1998) Development and testing of a bacterial biosensor for toluene-based environmental contaminants. Appl Environ Microbiol 64: 1006–1012. 9501440 10.1128/aem.64.3.1006-1012.1998 PMC106358

[29] Association AWW (1998) Standard Methods for the Estimation of Water and Wastewater (APHA). Washington, DC: Water Environment Federation. (http://www.standardmethods.org/store/BrowseSM.cfm?PartID=5. Accessed 2012 July 27).

[30] Lei Y, Chen W, Mulchandani A (2006) Microbial biosensors. Anal Chim Acta 568: 200–210. 17761261 10.1016/j.aca.2005.11.065

[31] Bronstein I, Fortin J, Stanley PE, Stewart GS, Kricka LJ (1994) Chemiluminescent and bioluminescent reporter gene assays. Anal Biochem 219: 169–181. 8080073 10.1006/abio.1994.1254

[32] Altschmied J, Duschl J (1997) Set of optimized luciferase reporter gene plasmids compatible with widely used CAT vectors. Biotechniques 23: 436–438. 9298214 10.2144/97233bm19

[33] Hakkila K, Maksimow M, Karp M, Virta M (2002) Reporter genes lucFF, luxCDABE, gfp, and dsred have different characteristics in whole-cell bacterial sensors. Anal Biochem 301: 235–242. 11814294 10.1006/abio.2001.5517

[34] Park SM, Park HH, Lim WK, Shin HJ (2003) A new variant activator involved in the degradation of phenolic compounds from a strain of Pseudomonas putida. J Biotechnol 103: 227–236. 12890609 10.1016/s0168-1656(03)00122-6

[35] Suresh PS, Kumar R, Kumar A (2010) Three Dimensional Model for N-Terminal A Domain of DmpR (2-Dimethylphenol) Protein Based on Secondary Structure Prediction and Fold Recognition. In Silico Biology 10: 223–233. 22430356 10.3233/ISB-2010-0434

[36] DeLano WL (2002) The PyMOL Molecular Graphics System Available at: http://www.pymol.org.Accessed 2012 July 27.

[37] Laskowski RA, Chistyakov VV, Thornton JM (2005) PDBsum more: new summaries and analyses of the known 3D structures of proteins and nucleic acids. Nucleic Acids Res 33: D266–268. 15608193 10.1093/nar/gki001 PMC539955

[38] Shingler V, Pavel H (1995) Direct regulation of the ATPase activity of the transcriptional activator DmpR by aromatic compounds. Mol Microbiol 17: 505–513. 8559069 10.1111/j.1365-2958.1995.mmi_17030505.x

[39] Forsman M, Romantschuk M, Sarand I, Ska E, Shingler V (2001) Role of the DmpR-Mediated Regulatory Circuit in Bacterial Biodegradation Properties in Methylphenol-Amended Soils. Society 67: 162–171 doi: 10.1128/AEM.67.1.162. 10.1128/AEM.67.1.162-171.2001 PMC92538 11133441

[40] Michałowicz J, Duda W (2007) Phenols – Sources and Toxicity. 16: 347–362.

[41] Escher BI, Snozzi M, Häberli K, Schwarzenbach RP (1997) A new method for simultaneous quantification of uncoupling and inhibitory activity of organic pollutants in energy-transducing membranes. Environmental Toxicology and Chemistry 16: 405–414.

[42] Escher BI, Snozzi M, Schwarzenbach RP (1996) Uptake, Speciation, and Uncoupling Activity of Substituted Phenols in Energy Transducing Membranes. Environmental Science & Technology 30: 3071–3079.

[43] Kelsey JW, Kottler BD, Alexander M (1996) Selective chemical extractants to predict bioavailability of soil-aged organic chemicals environmental Science & Technology. 31: 214–217.

[44] Kohler S, Belkin S, Schmid RD (2000) Reporter gene bioassays in environmental analysis. Fresenius J Anal Chem 366: 769–779. 11225788 10.1007/s002160051571

